# Meteorological Factors and the Transmissibility of Hand, Foot, and Mouth Disease in Xiamen City, China

**DOI:** 10.3389/fmed.2020.597375

**Published:** 2021-01-22

**Authors:** Jingwen Xu, Meng Yang, Zeyu Zhao, Mingzhai Wang, Zhinan Guo, Yuanzhao Zhu, Jia Rui, Yao Wang, Xingchun Liu, Shengnan Lin, Li Luo, Yanhua Su, Benhua Zhao, Yulin Zhou, Roger Frutos, Tianmu Chen

**Affiliations:** ^1^State Key Laboratory of Molecular Vaccinology and Molecular Diagnostics, School of Public Health, Xiamen University, Xiamen City, China; ^2^Xiamen Center for Disease Control and Prevention, Xiamen City, China; ^3^United Diagnostic and Research Center for Clinical Genetics, Women and Children's Hospital, School of Medicine & School of Public Health, Xiamen University, Xiamen City, China; ^4^Agricultural Research Centre for International Development, Intertryp, Montpellier, France; ^5^Institut d'Electronique et des Systèmes, Université de Montpellier-Centre National de la Recherche Scientifique, Montpellier, France

**Keywords:** infectious disease, hand, foot, mouth disease, seasonality, transmissibility, meteorological factors

## Abstract

**Background:** As an emerging infectious disease, the prevention and control of hand, foot, and mouth disease (HFMD) poses a significant challenge to the development of public health in China. In this study, we aimed to explore the mechanism of the seasonal transmission characteristics of HFMD and to reveal the correlation and potential path between key meteorological factors and the transmissibility of HFMD.

**Methods:** Combined with daily meteorological data such as average temperature, average relative humidity, average wind velocity, amount of precipitation, average air pressure, evaporation capacity, and sunshine duration, a database of HFMD incidence and meteorological factors was established. Spearman rank correlation was used to calculate the correlation between the various meteorological factors and the incidence of HFMD. The effective reproduction number (*R*_*eff*_) of HFMD was used as an intermediate variable to further quantify the dynamic relationship between the average temperature and *R*_*eff*_.

**Results:** A total of 43,659 cases of HFMD were reported in Xiamen from 2014 to 2018. There was a significantly positive correlation between the average temperature and the incidence of HFMD (*r* = 0.596, *p* < 0.001), and a significantly negative correlation between the average air pressure and the incidence of HFMD (*r* = −0.511, *p* < 0.001). There was no correlation between the average wind velocity (*r* = 0.045, *p* > 0.05) or amount of precipitation (*r* = 0.043, *p* > 0.05) and incidence. There was a temperature threshold for HFMD's transmissibility. Owing to the seasonal transmission characteristics of HFMD in Xiamen, the temperature threshold of HFMD's transmissibility was 13.4–18.4°C and 14.5–29.3°C in spring and summer and in autumn and winter, respectively.

**Conclusions:** HFMD's transmissibility may be affected by the average temperature; the temperature threshold range of transmissibility in autumn and winter is slightly wider than that in spring and summer. Based on our findings, we suggest that the relevant epidemic prevention departments should pay close attention to temperature changes in Xiamen to formulate timely prevention strategies before the arrival of the high-risk period.

## Introduction

Hand, foot, and mouth disease (HFMD) is an infectious viral disease caused by coxsackievirus A16 (CVA16) and enterovirus 71 (EV71) and other enteroviruses (1, 2). It was first reported in New Zealand in 1957 and is a common global infantile infectious disease (3). The disease occurs in infants under 5 years old and is characterized by stomatalgia, anorexia, low fever, skin, or mucosal ulcers of the hands, feet, and mouth. However, a small number of children may experience myocarditis, pulmonary oedema, aseptic meningoencephalitis, and other complications. EV71 is often associated with serious central nervous system diseases and other complications, and the disease can develop rapidly in some severe cases, even leading to death. Since the discovery of EV71 (in Vietnam) in 1969, there have been HFMD outbreaks of varying degrees throughout the Asia-Pacific region, including China (4). In a populous country such as China, an average of approximately 2 million cases of HFMD are reported every year. Thirty-one provinces across the country have reported cases of HFMD infection. HFMD is one of the top three class C notifiable infectious diseases with the highest incidence and highest number of deaths in China. Although the first EV71 vaccine in China was approved by the Chinese Food and Drug Administration (CFDA) at the end of 2015, the safety and effectiveness of the vaccine has not been evaluated in a wide range of people for an extended period. Thus, the effectiveness of the EV71 vaccine is limited (5–7). Therefore, in addition to continuing to develop multivalent vaccines, epidemic prevention departments should pay more attention to the potential factors and transmission mechanisms that cause outbreaks of HFMD.

Many studies have shown that the spread of HFMD has complex seasonality, that some areas have a multi-peak epidemic, and that the time rhythm of the epidemic peak varies with latitude. For example, the epidemic peak of HFMD occurs twice a year in subtropical areas such as Hong Kong (8) and Taiwan (9), and in tropical regions such as Vietnam, Singapore, and Malaysia (10). In contrast, there are unimodal epidemic characteristics in high latitude areas such as Japan (11) and Finland (12). In northern China, HFMD reaches its peak in summer while HFMD in the south reaches its peak in spring and autumn (13, 14). These countries show different HFMD epidemiological characteristics, and the various differences show that the outbreak and prevalence of infectious diseases have a series of potential influencing factors (15), such as natural factors, socio-economic factors, and regional policies (16, 17). These may have an interactive impact on the transmission characteristics of the disease. Among the health effects caused by climate change, one of the most important aspects is the impact on the spread of infectious diseases, and the intervention of pathogens, hosts, transmission route, and other intermediate links first reflects the mode of action of meteorological variables, which ultimately affect the incidence of the disease. Currently, there is an urgent need to solve the “black box” problem in this process, i.e., in what manner and what intervention role do a series of meteorological variables play in affecting the incidence of HFMD? Thus far, there have been relatively few studies and direct evidence on this issue.

In China, the annual incidence rate in the southern and eastern provinces is higher than that of other provinces, and the annual incidence rate in Fujian Province is reported to be higher than the national average. The incidence of HFMD in Xiamen is always at the forefront in Fujian Province. Therefore, it is necessary to perform timely prevention and control measures for HFMD. In this study, the effective reproduction number (*R*_*eff*_) obtained by mathematical model fitting was used to quantify the transmissibility of HFMD and as an intermediate variable to explore the relationship between key meteorological factors and HFMD. This can effectively restore the action mechanism of meteorological factors, and help establish preventive measures and an early warning mechanism for HFMD in the study area by combining real events.

## Materials and Methods

### Study Area

**Figure 1:**
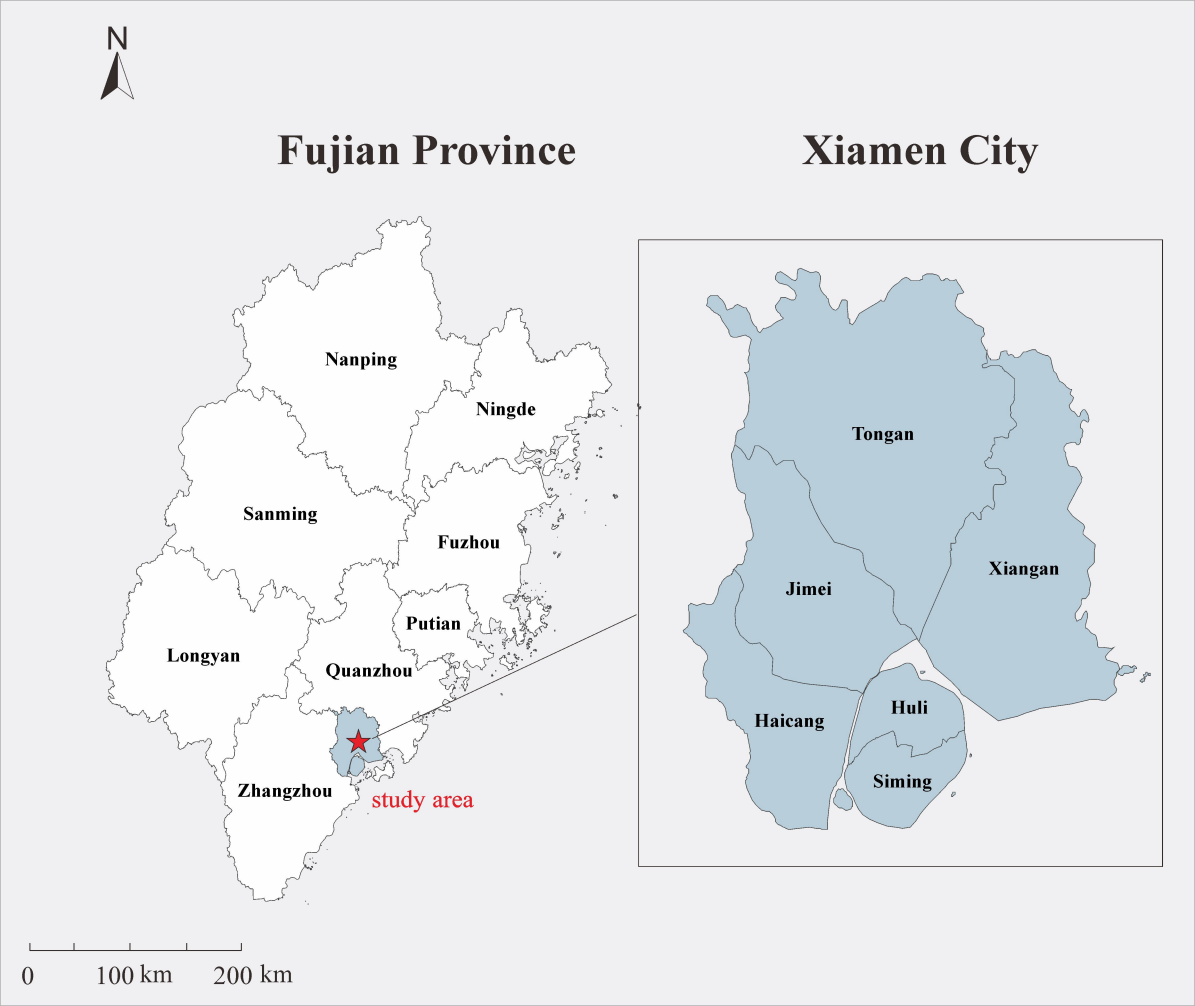
Geographical location of the study area. The map depicted in this figure was taken from Wikimedia Commons (http://commons.wikimedia.org/wiki/Main_Page).

Xiamen was selected as the study area as it is a special economic zone in China approved by the State Council, as well as an important central city, port, and scenic tourist area along the southeast coast. Xiamen is situated along the north latitude of 24°23′ N to 24°54′ N and east longitude of 117°53′ E to 118°26′ E in the south of Fujian Province, connected to Zhangzhou and Quanzhou cities (Figure 1). The climate is a subtropical marine monsoon climate, with mild and rainy conditions. The annual average temperature is ~21°C. There is no severe cold weather in winter and no heat in summer. The annual average precipitation is mainly concentrated from April to August, and decreases from northwest to southeast. As it is located on the coast, Xiamen is windy, with high wind speeds. In 2018, the residential population of Xiamen was 4.11 million (according to the Xiamen Bureau of Statistics).

### Data on HFMD

In this study, we selected cases of HFMD reported by the China Disease Prevention and Control Information System in Xiamen from January 2014 to December 2018. The illness onset date of each case was collected. The diagnosis of HFMD is based on the diagnostic criteria of *The Guidelines for the Diagnosis and Treatment of HFMD* (*2010 Edition*) issued by the National Health Commission (formerly the “Ministry of Health”) of the People's Republic of China (18). All cases should be considered in combination with the epidemiological history, clinical manifestations, and etiological examination. Based on the clinical diagnosis, the patient can be diagnosed as a confirmed case according to any of the following conditions: positive enterovirus specific nucleic acid test, enterovirus isolation, identified as CV-A16, EV-A71, or other enteroviruses that can cause HFMD, and acute phase serum-related virus IgM antibody positive. Severe cases of HFMD are clinical manifestations, such as nervous system involvement and respiratory and circulatory system dysfunction.

### Meteorological Parameters

The data required for this study were selected from the meteorological data reported by the Xiamen Meteorological Bureau from 2014 to 2018, including the average temperature, average relative humidity, average wind velocity, amount of precipitation, average air pressure, evaporation capacity, sunshine duration, and other meteorological factors. For diseases with a short incubation period, the use of the daily exposure in studies can provide sufficient time for health authorities to prepare for possible outbreaks. Therefore, we collected meteorological factor data on a daily basis and established a meteorological factor observation database for the entire process of research and analysis process (19).

### Data Analysis

**Figure 2:**
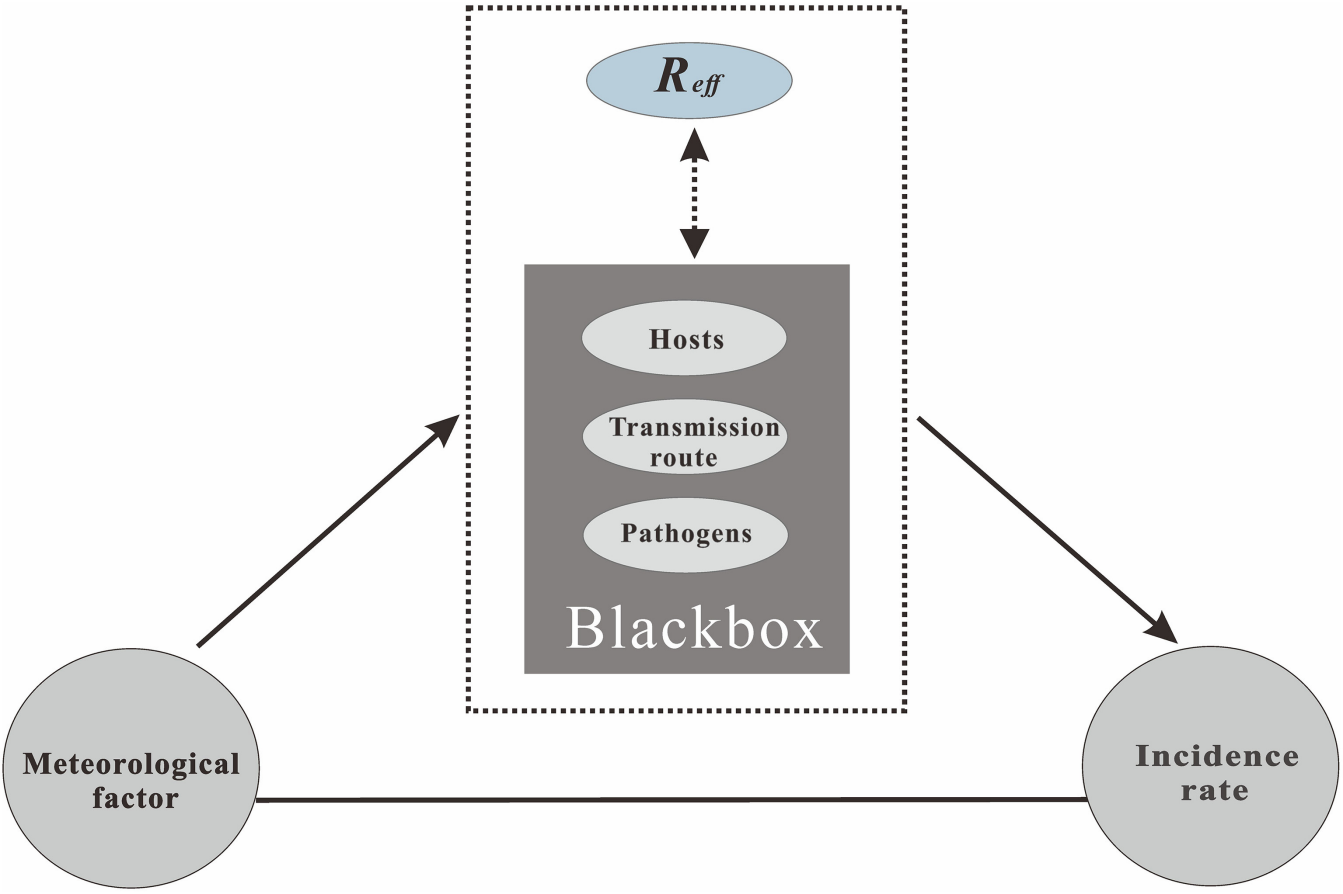
The process of the meteorological factors that affect the incidence of infectious diseases.

In this study, we assumed that meteorological factors first act on an intermediate medium related to HFMD, which then affects the characteristics of the disease through changes in the intermediate medium. There are three main ways (i.e., pathogens, hosts, or the transmission route of the disease) in which meteorological factors affect the disease hosts (20–22). Figure 2 shows the complete path of action. Thus, the data analysis in the study was divided into two stages. The first stage was the analysis of the relationship between the collected meteorological factors and the daily incidence rate, accounting for the delay in the incubation period and diagnosis of HFMD. In the correlation analysis, according to the incubation period of HFMD (3–7 days), we selected a lag of 10 days as the maximum lag in the incidence of HFMD. Thus, the number of cases was adjusted by a lag of 0–10 days. Considering that the selected data are not equidistant continuous data, and that the Pearson product moment correlation coefficient has limitations in its use, the non-parametric Spearman rank correlation coefficient was introduced in this study, which can be calculated as follows:

(1) $$\begin{array}{r} \rho_{S}=\frac{Cov\left(F\left(X_{1}\right),F\left(X_{2}\right)\right)}{\sqrt{Var\left(F\left(X_{1}\right)\right)}\sqrt{Var\left(F\left(X_{2}\right)\right)}}\\ \;=\frac{E\left(F\left(X_{1}\right)-E\left(F\left(X_{1}\right)\right)\right)\left(F\left(X_{2}\right)-E\left(F\left(X_{2}\right)\right)\right)}{\sqrt{Var\left(F\left(X_{1}\right)\right)}\sqrt{Var\left(F\left(X_{2}\right)\right)}} \end{array}$$

According to the optimal value of the Spearman rank correlation analysis, the main meteorological factors affecting the incidence of HFMD were selected, and the data were analyzed by SPSS v. 21.0 (IBM Company, U.S.A.). A *P-*value of < 0.05 was considered statistically significant. The degree of collinearity between independent variables was observed based on the correlation analysis. If there was strong collinearity (*r* > 0.7), stepwise regression analysis was conducted to eliminate independent variables. In the second stage of our analysis, the correlation between the main meteorological factors and the *R*_*eff*_ of HFMD was analyzed. According to the *R*_*eff*_ epidemic curve, we determined each time node of the correlation analysis to restore the correlation between the meteorological factors and *R*_*eff*_ in each time period. In our study, the relevant parameters were obtained from the seasonal study of HFMD in Xiamen (23).

A susceptible–exposed–infectious–asymptomatic–removed (SEIAR) model was established based on the natural history of HFMD. The SEIAR model divided people into the five following categories: susceptible individuals (S), exposed, but not yet infected individuals (E), infectious and symptomatic individuals (I), asymptomatic individuals (A), which indicates infectious, but not symptomatic, and recovered individuals (R). There were nine parameters in the SEIAR model: relative transmission rate (β), relative transmissibility rate of asymptomatic to symptomatic individuals (κ), proportion of symptomatic (*p*), incubation relative rate (ω), recovery rate of infectious (γ), recovery rate of asymptomatic (γ'), fatality rate of HFMD cases (*f* ), birth rate (*br*), and natural death rate of the entire city (*dr*).

The *R*_*eff*_, which was defined as the number of secondary cases infected by the first case during his or her infectious period, was used to quantify the transmissibility of HFMD (24). The corresponding calculation formula in the SEIAR model is as follows:

(2) $$\begin{array}{r} R_{eff}=\beta S\left(\frac{1-p}{\gamma }+\frac{\kappa p}{\gamma^{\prime }}\right), \end{array}$$

The seasonality of the *R*_*eff*_ can be simulated with the following trigonometric function:

(3) $$\begin{array}{r} R_{eff\left(t\right)}=\beta_{0}S\left(\frac{1-p}{\gamma }+\frac{\kappa p}{\gamma^{\prime }}\right)\left[1+\sin\left(\frac{2\pi \left(t+\alpha \right)}{T}\right)\right], \end{array}$$

where β_0_, *t*, α, and *T* refer to the baseline of the transmission relative rate, time, a constant, which adjusts the position of time, and the time span of the season cycle, respectively. Details on the SEIAR model, the values of the parameters, and the seasonal values of *R*_*eff*_ have been previously published (23).

## Results

### Incidence and Transmissibility of HFMD

A total of 43,659 cases of HFMD were collected from 1 January 2014 to 31 December 2018. The results showed that both the *R*_*eff*_ and incidence had notable periodicity, as there were two peaks in 1 year, and the peak incidence lagged behind the peak *R*_*eff*_value.

### Incidence of HFMD and Meteorological Factors

**Figure 3:**
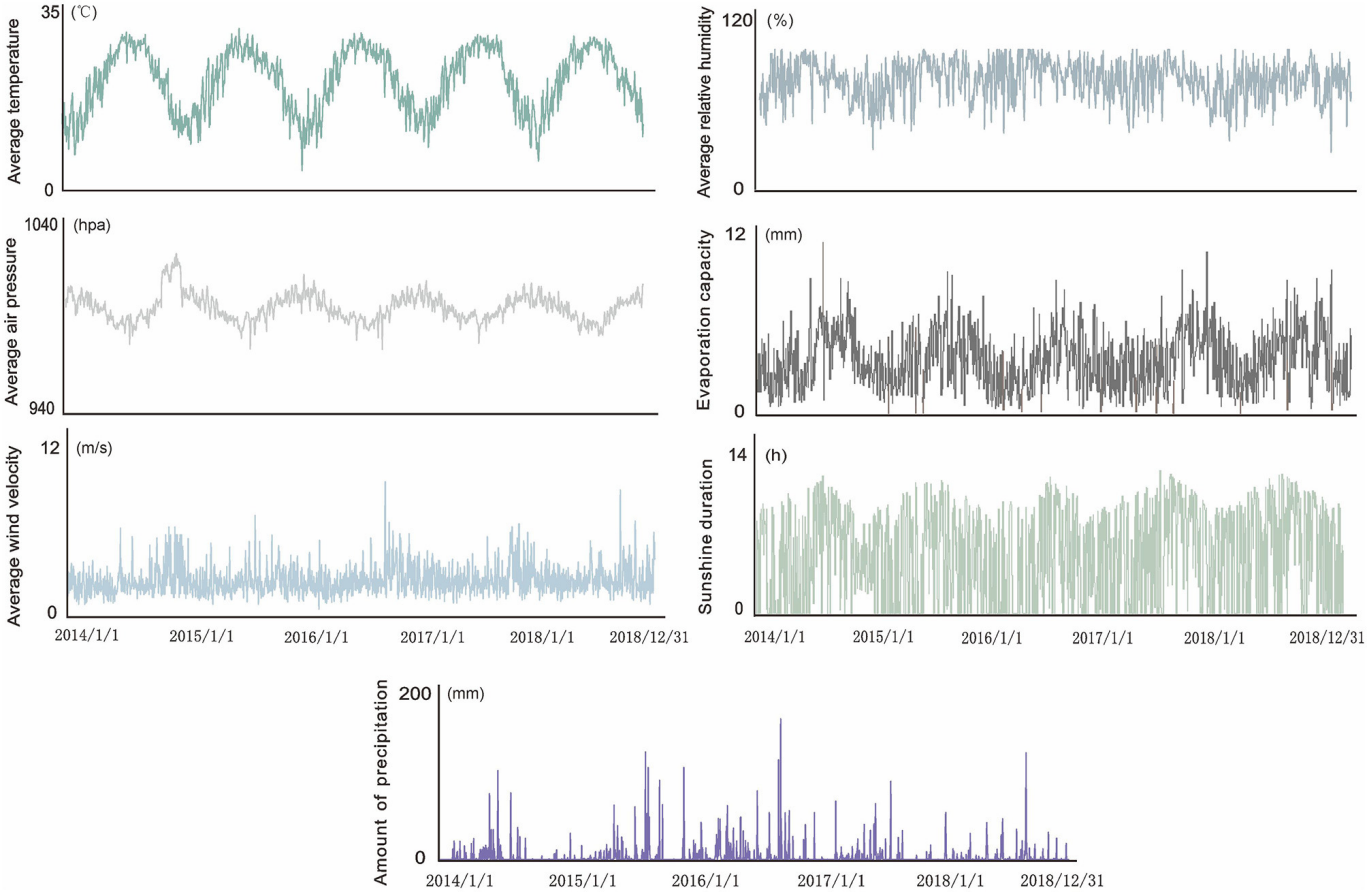
Daily variation in meteorological factors from 2014 to 2018.

The highest daily temperature for the study period was 38.5°C, the lowest was 0.1°C, and the average temperature was 21.5°C. Precipitation ranged between 0 and 172.7 mm, daily sunshine duration ranged between 0 and 13 h, average relative humidity between 27 and 99%, average air pressure between 974.6 and 1,017.2 hPa, and evaporation capacity between 0 and 9.9 mm (Figure 3). Based on the incidence epidemic curve, the peak of incidence was mainly concentrated in summer and autumn, where the peak in summer was slightly lower than that in autumn. However, an abnormal situation occurred in 2017, when the peak in summer was significantly higher than that in autumn. The Spearman rank correlation analysis (Table 1) showed a significantly positive correlation between the average temperature and incidence of HFMD (*r* = 0.596, *p* < 0.001), and a significantly negative correlation between the average air pressure and the incidence of HFMD (*r* = −0.511, *p* < 0.001). There was no correlation between the average wind velocity or amount of precipitation and incidence. Considering that the influence of lag may have distorted the results of the correlation analysis, the lag time of each meteorological variable was set to 0–10 days. After the adjustment, the correlations were slightly enhanced compared to those of the initial results. There was a weak correlation between the average wind velocity, amount of precipitation, and incidence, but overall, the results did not change significantly. The second set of results showed that there was a strong correlation between the average temperature and average air pressure (*r* = −0.841, *p* < 0.001). Therefore, these meteorological parameters and incidence rate were included in a multiple linear regression model (F_in_ > F_α_). The model indicated that the average air pressure should be eliminated. The multiple correlation coefficient was 0.527, and the significance test of regression was *P* < 0.05, indicating that the degree of regression was significant.

**Table 1:** Correlation analysis between the meteorological factors and the incidence of hand, foot, and mouth disease (HFMD) in Xiamen City, China.

**Variant**	**Lag0**		**Lag1**		**Lag2**		**Lag3**		**Lag4**		**Lag5**		**Lag6**		**Lag7**		**Lag8**		**Lag9**		**Lag10**	
	***r***	***p***	***r***	***p***	***r***	***p***	***r***	***P***	***r***	***p***	***r***	***p***	***r***	***p***	***r***	***p***	***r***	***p***	***r***	***p***	***r***	***p***
Average temperature	0.596	<0.001	0.592	<0.001	0.591	<0.001	0.596	<0.001	0.601	<0.001	0.601	<0.001	0.599	<0.001	0.594	<0.001	0.592	<0.001	0.588	<0.001	0.585	<0.001
Average relative humidity	0.176	<0.001	0.179	<0.001	0.189	<0.001	0.211	<0.001	0.234	<0.001	0.250	<0.001	0.256	<0.001	0.246	<0.001	0.239	<0.001	0.237	<0.001	0.232	<0.001
Average wind velocity	0.045	>0.05	0.052	<0.05	0.051	<0.05	0.041	>0.05	0.023	>0.05	0.016	>0.05	0.003	>0.05	0.001	>0.05	0.006	>0.05	−0.003	>0.05	0.002	>0.05
Amount of precipitation	0.043	>0.05	0.036	>0.05	0.042	>0.05	0.058	<0.05	0.066	<0.05	0.065	<0.05	0.071	<0.05	0.076	>0.05	0.082	<0.001	0.079	>0.05	0.076	>0.05
Average air pressure	−0.511	<0.001	−0.508	<0.001	−0.510	<0.001	−0.522	<0.001	−0.534	<0.001	−0.54	<0.001	−0.544	<0.001	−0.543	<0.001	−0.543	<0.001	−0.542	<0.001	−0.544	<0.001
Evaporation capacity	0.258	<0.001	0.263	<0.001	0.266	<0.001	0.247	<0.001	0.223	<0.001	0.210	<0.001	0.199	<0.001	0.205	<0.001	0.207	<0.001	0.205	<0.001	0.207	<0.001
Sunshine duration	0.130	<0.001	0.125	<0.001	0.120	<0.001	0.103	<0.001	0.093	<0.001	0.091	<0.001	0.084	<0.001	0.086	<0.001	0.079	<0.05	0.082	<0.001	0.084	<0.001

### Transmissibility of HFMD and Meteorological Factors

**Figure 4:**
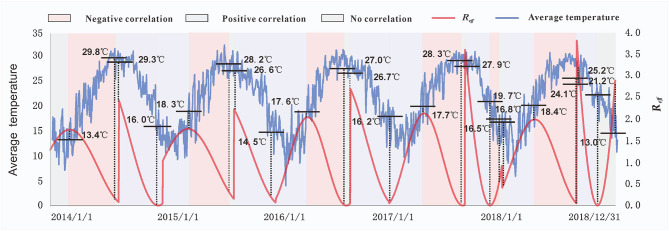
The relationship between the effective reproduction number (*R*_*eff*_) and average temperatures. The temperature values marked in the figure represent the temperatures corresponding to each key node of the *R*_*eff*_. Different background colors indicate the correlation between the *R*_*eff*_ and average temperature at different stages. Purple represents a positive correlation, pink represents a negative correlation, and gray represents no correlation.

**Figure 5:**
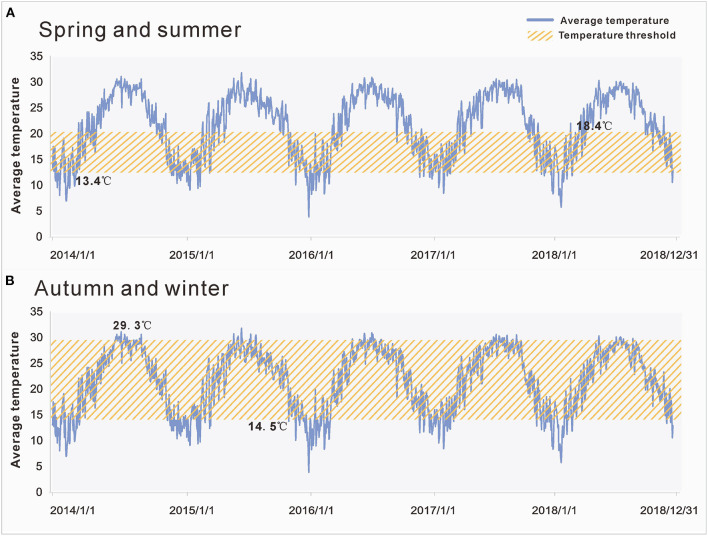
Temperature thresholds of effective reproduction number (*R*_*eff*__)_ in different seasons. **(A)** The temperature threshold of *R*_*eff*_ in spring and summer was 13.4–18.4°C. **(B)** The temperature threshold of *R*_*eff*_ in autumn and winter was 14.5–29.3°C. The yellow area represents the range covered by the temperature threshold.

Based on the main meteorological factors screened out in the first stage of our analysis, we further explored the relationship between the average temperature and *R*_*eff*_. Preliminary observations showed that when *R*_*eff*_ was lower or higher than a certain temperature threshold, *R*_*eff*_ changed in the opposite direction of the average temperature curve. We assumed that this phenomenon was because the spread of enteroviruses requires certain temperature conditions; thus, the growth and reproduction of enteroviruses will be limited if they are below or above a certain temperature range. Therefore, at this stage of the analysis, the Spearman rank correlation was used to analyse the correlation between the average temperature and *R*_*eff*_ in each period (Figure 4). As the peak incidence of HFMD occurred in Xiamen twice a year, it had significant seasonal characteristics. Therefore, our results presented two situations according to the season (Figure 5): in spring and summer, the temperature threshold of *R*_*eff*_ was 13.4–18.4°C, whereas in autumn and winter, the temperature threshold of *R*_*eff*_ was 14.5–29.3°C.

## Discussion

Although some studies focus on the relationship between meteorological factors and incidence of HFMD, our study is the first to explore the relationship between the meteorological factors and the transmissibility of this disease. In studies on the short-term influence that meteorological variables have on HFMD, the commonly used method is model analysis including non-linearity or lag. Although this type of model can partially avoid the influence of confounding factors, it has not completely restored the complete action path of meteorological factors.

Recently, 31 provinces, autonomous regions, and municipalities directly under the central government have suffered from HFMD. Xiamen is located on the southeast coast, a famous tourist city, with a subtropical marine monsoon climate, and mild and rainy conditions throughout the year, which is representative in meteorological research.

In this study, we found that the incidence of HFMD in Xiamen had a more notable seasonal characteristic. There were two peaks in the year, with the peak in autumn generally slightly higher than that in summer. In 2017, the peak incidence of HFMD in summer was significantly higher than that in autumn, which was partially related to the increase in the clustering epidemic situation in summer. At the same time, the incidence in autumn was lower than that in previous years, which may have been related to the reduction in the number of foreign tourists at this stage, which was due to a variety of factors, such as restrictions on tourist attractions or changes in traffic control measures (25).

Research on the seasonal characteristics of HFMD has been relatively thorough. Some studies have highlighted the importance of the effect that meteorological factors have on the seasonal distribution of HFMD (19, 26); however, the key meteorological factors obtained from different studies are different, and, even if they are the same, they differ in their relationship with HFMD. This difference was mainly affected by the geographical heterogeneity. Therefore, to obtain more accurate and detailed findings, future research should focus on specific study areas.

The seasonal transmission characteristics of infectious diseases are closely related to the infectivity of pathogens (26), human activities, human susceptibility, and other mechanisms. Meteorological factors play a role by promoting or suppressing these mechanisms. HFMD can be spread through the gastrointestinal tract or respiratory tract (such as spraying, coughing, and sneezing). When the climate in Xiamen changes from spring to summer, the frequency of children's outings and outdoor sports (such as swimming) increases, which increases the probability of contact between people (27). At the same time, indoor or public places are often enclosed to maintain warm temperatures, ignoring the importance of ventilation, which will increase exposure to pathogens in the air and increase the risk of HFMD. When Xiamen transitions from summer to autumn, the climate is relatively cool, people often conduct family oriented short-distance tourism during this period, and some local scenic spots have large-scale crowd gatherings. In addition, kindergartens, primary schools, and other educational institutions are at their peak school opening in autumn, and school opening times are relatively concentrated, which makes collective infection more feasible during the epidemic period of HFMD. In our study, the peak value of HFMD in autumn was slightly higher than that in summer, which was related to the large-scale inflow of foreign tourists into Xiamen. Xiamen, a city with a well-developed tourism industry, has large numbers of tourists at different stages every year. However, autumn is the peak tourism period, when foreign tourists can play the role of pathogen carriers, spreading pathogens and viruses to areas that do not have infectious diseases, resulting in an epidemic. Additionally, the visiting population (28) may have a temporary maladjustment to the sudden change in geographical environment and eating habits, resulting in a decline in their immunity, making them more susceptible to infectious diseases. Thus, the seasonal drivers of infectious diseases are highly complex. In addition to some of the main factors discussed above, the seasonal pattern of HFMD may also be related to social and economic policies (29) and other factors, which require further exploration.

Meteorological factors have a lag effect on the incidence of HFMD. This is because they first act on the intermediate medium and then affect the final incidence of HFMD through the intermediate medium. This intermediate process must complete its cycle over a certain period. In this study, there was no significant change in the results of the correlation analysis between the weather and HFMD after adjustments for the lag phenomenon, indicating that the lag of the disease had an impact on the correlation analysis but had no decisive impact on the final results. Most studies have found that one or more meteorological factors are related to HFMD (9, 30–32), and our study draws a similar conclusion. However, the ways in which meteorological factors affect the occurrence of infectious diseases and their innate path of action are rarely discussed. We suggest that theoretical epidemiology is an important means to reveal this “intermediate chain.” In this seasonal study on HFMD in Xiamen, *R*_*eff*_ was used to measure the transmissibility of HFMD. Under the premise that the average temperature was the main factor affecting the disease, we further explored the relationship between the *R*_*eff*_ and average temperature to observe the action pathway of meteorological factors. Our findings showed that the transmissibility of the virus increased with the increase in temperature, but decreased rapidly after reaching a temperature threshold, after which there was a decrease in virus transmissibility. This indicates that temperature has a significant influence on the survival and transmission of enteroviruses in the external environment and in hosts as well as on the behavior of hosts. The sensitivity of EV71 and CVA16 to temperature has been confirmed in previous studies (33, 34). Furthermore, studies have shown that the increment efficiency and speed of EV71 at 32, 35, and 40°C are inhibited compared with that at 37°C (35). Moreover, temperature sensitivity is the decisive factor for the survival of the virus in hosts. For example, the temperature-sensitive mutant of EV71 is less infectious and pathogenic to hosts than the temperature-tolerant mutant, which limits the transmission of the virus between hosts (36). In other studies, temperature can not only directly affect the trajectory of human activities but also have a great impact on the behavior and physical condition of the human body (37). Therefore, the changing characteristics of the *R*_*eff*_ of HFMD reflected the comprehensive effect of a variety of intermediate media.

In summary, our analysis partially revealed the direct impact that the main meteorological factors have on the onset of HFMD, which mitigated the potential impact of the incubation period and treatment time on our research findings to the greatest extent possible. The meteorological and HFMD transmissibility data used in this study focussed only on Xiamen City; thus, the data source was limited, which led to our study not being more widely representative. In future research, we can consider nationwide data collection to further explore the main meteorological factors related to the transmissibility of HFMD. Related studies have also shown that the main pathogens of HFMD change in different seasons and years, as well as a high mutation rate of human enterovirus (38, 39). Besides, as current research on the temperature threshold of transmissibility is relatively limited, there is no unified threshold judgment standard. At present, this study is unable to effectively solve these problems. To obtain more practical research results, these aspects should be included in further explorations of the methods.

## Conclusions

Our results showed that there was a high correlation between the average temperature and incidence of HFMD, as well as potential temperature thresholds for the transmissibility of HFMD. We suggest that close attention should be paid to temperature factors in the process of prevention and control of HFMD in Xiamen. Temperature changes should be incorporated into an early warning mechanism for HFMD, and timely preventive measures should be taken before the arrival of the high-risk period.

## Data Availability Statement

The raw data supporting the conclusions of this article will be made available by the authors, without undue reservation.

## Author Contributions

TC, YZho, RF, JX, and MY designed the study. JX, MY, ZZ, MW, ZG, and YZhu collected the data. JR, XL, ZZ, SL, LL, BZ, YS, and TC analyzed the data. TC, YZ, RF, JX, and ZZ wrote the manuscript. All authors have read and approved the final manuscript.

## Conflict of Interest

The authors declare that the research was conducted in the absence of any commercial or financial relationships that could be construed as a potential conflict of interest.
